# *Lactobacillus rhamnosus* HN001 facilitates the efficacy of dual PI3K/mTOR inhibition prolonging cardiac transplant survival and enhancing antitumor effect

**DOI:** 10.1128/spectrum.01839-23

**Published:** 2024-04-02

**Authors:** Xiaolong Miao, Peng Jiang, Xiaotong Zhang, Xinqiang Li, Zelai Wu, Yuancong Jiang, Han Liu, Weixun Xie, Xinwei Li, Bingfeng Shi, Jinzhen Cai, Weihua Gong

**Affiliations:** 1 Organ Transplantation Center, The Affiliated Hospital of Qingdao University, Qingdao, China; 2 Department of Surgery, Second Affiliated Hospital of School of Medicine, Zhejiang University, Hangzhou, China; 3 Medical department, Qingdao Eighth People’s Hospital, Qingdao, China; 4 Department of Chemistry, Zhejiang University, Hangzhou, Zhejiang, China; 5 Liangzhu Laboratory, Zhejiang University Medical Center, Hangzhou, China; Huazhong University of Science and Technology, Wuhan, China

**Keywords:** allograft rejection, primary liver cancer, PI3K/mTOR dual inhibitor, *Lactobacillus rhamnosus *HN001

## Abstract

Solid organ transplantation is a crucial treatment for patients who have reached the end stage of heart, lung, kidney, or liver failure. However, the likelihood of developing cancer post-transplantation increases. Additionally, primary malignant tumors remain a major obstacle to the long-term survival of transplanted organs. Therefore, it is essential to investigate effective therapies that can boost the immune system’s ability to combat cancer and prevent allograft rejection. We established a mouse orthotopic liver tumor model and conducted allogeneic heterotopic heart transplantation. Various treatments were administered, and survival curves were generated using the Kaplan–Meier method. We also collected graft samples and measured inflammatory cytokine levels in the serum using an inflammatory array. The specificity of the histochemical techniques was tested by staining sections. We administered a combination therapy of phosphoinositide 3-kinase/mammalian target of rapamycin (PI3K/mTOR) dual inhibitor BEZ235 and *Lactobacillus rhamnosus* HN001 to primary liver cancer model mice with cardiac allografts. Consistent with our prior findings, *L. rhamnosus* HN001 alleviated the intestinal flora imbalance caused by BEZ235. Our previous research confirmed that the combination of BEZ235 and *L. rhamnosus* HN001 significantly prolonged cardiac transplant survival.

**IMPORTANCE:**

We observed that the combination of phosphoinositide 3-kinase/mammalian target of rapamycin (PI3K/mTOR) dual inhibitor BEZ235 and *Lactobacillus rhamnosus* HN001 notably prolonged cardiac transplant survival while also inhibiting the progression of primary liver cancer. The combination therapy was efficacious in treating antitumor immunity and allograft rejection, as demonstrated by the efficacy results. We also found that this phenomenon was accompanied by the regulation of inflammatory IL-6 expression. Our study presents a novel and effective therapeutic approach to address antitumor immunity and prevent allograft rejection.

## INTRODUCTION

Due to the declined incidence of acute rejection with the progress of immunosuppressive therapy, short-term graft survival after solid organ transplantation has dramatically improved (1). Nevertheless, the risk of cancer among solid organ transplant recipients is two to four times higher in comparison to general population (2–6). Malignancy is a major cause of morbidity and mortality following heart transplantation (7). Therefore, it is urgent to identify novel and effective treatments for solid organ transplant recipients.

Immunosuppressive therapy after transplantation significantly increases the risk of solid tumors (especially liver cancer) in solid organ transplant recipients (8, 9). Furthermore, some immunosuppressive drugs, such as calcineurin inhibitors and azathioprine, facilitate carcinogenesis through mechanisms that are distinct from their immunosuppressive effects (10, 11). Contrarily, novel therapeutic agents, such as mycophenolate mofetil and mammalian target of rapamycin (mTOR) inhibitors, may exhibit antineoplastic effects in addition to immunosuppression (11, 12). PI3K/mTOR dual inhibitor BEZ235, an imidazo(4,5-c) quinoline derivative, exerts inhibitory effects on phosphoinositide 3-kinase (PI3K) (p110-α, -β, -γ, and -δ isoforms) as well as mTOR kinase activity, through binding to the ATP-binding cleft of these enzymes (13). Our previous study result indicates that BEZ235 is able to effectively prolong cardiac transplant survival in animal model (14). Furthermore, our research has revealed that BEZ235 also exhibits promising therapeutic effects on solid tumors (15, 16).

The intestinal microbiota (IM) plays a crucial role in numerous physiological processes, including nutrient absorption and substrate metabolism (17). Additionally, the IM plays a pivotal role in modulating systemic immune responses (18–20). Given that the composition of the IM significantly impacts host immunity, achieving a balanced IM is imperative. Probiotics have demonstrated significant clinical benefits in manipulating the intestinal ecosystem to improve host-immune responses (21). *Lactobacillus rhamnosus* HN001 is a safe probiotic strain that exerts anti-inflammatory effects and modulates host immunity, thereby conferring health-enhancing benefits (22–24). This strain exhibits specific functions that relate to gut barrier integrity (25), microbial structure (26), and host metabolism (27). Supplementation with *L. rhamnosus* HN001 has been shown to significantly improve the balance and reduce intestinal inflammation (28).

Our findings indicate that the administration of *L. rhamnosus* HN001 can effectively counteract the intestinal dysbiosis caused by BEZ235 in transplant recipient mice (29). Furthermore, the concomitant treatment with BEZ235 and *L. rhamnosus* HN001 significantly prolongs cardiac transplant survival (29). Notably, the simultaneous administration of the PI3K/mTOR dual inhibitor BEZ235 and *L. rhamnosus* HN001 demonstrated a consistent increase in the duration of survival of cardiac transplant recipients, while concurrently suppressing the progression of primary liver cancer. The findings of the efficacy results of combination therapy revealed its potential in enhancing antitumor immune response and mitigating allograft rejection. In this study, we have introduced a new and effective approach for the treatment of antitumor immune response and allograft rejection.

## RESULTS

### Construction of a cardiac allograft-bearing primary liver cancer mouse model

**Fig 1:**
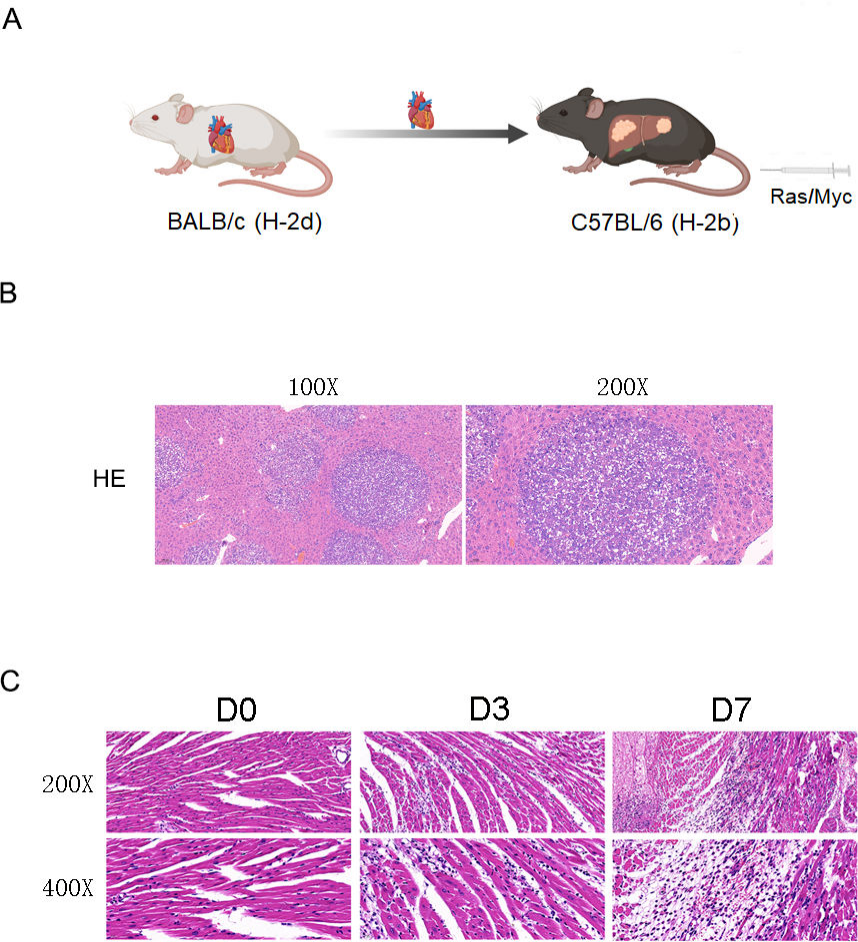
Construction of a cardiac allograft-bearing primary liver cancer mouse model. (**A**) The modeling process is illustrated in the schematic. (**B**) Histological analysis was performed on liver sections with original magnifications of 100× and 200×. (**C**) Histological analysis was carried out on the allografts. Heart transplant recipients were sacrificed on days 0, 3, and 7 following transplantation surgery, and the sections were viewed at original magnifications of 200× and 400×.

The methodology for modeling is illustrated in Fig. 1A. A murine animal model of primary liver cancer was established through hydrodynamic tail vein injection (HTVi) with murine transgenic constructs including pT/Caggs-NRas-V12, pT3-EF1a-C-Myc, and pCMVSB11, utilizing a prior method (15). Subsequently, an acute heterotopic cardiac transplantation rejection model was established in allogenic mice by transplanting hearts into their necks using the method outlined in a previous study (30). Liver tumor nodules were identified by hematoxylin-eosin (H&E) staining (Fig. 1B), confirming the establishment of the primary liver cancer model. The allografts were histologically evaluated over time post-transplantation (Fig. 1C), demonstrating a gradual increase in lymphocyte infiltration.

### Effect of BEZ235 and *Lactobacillus rhamnosus* HN001 in mice

**Fig 2:**
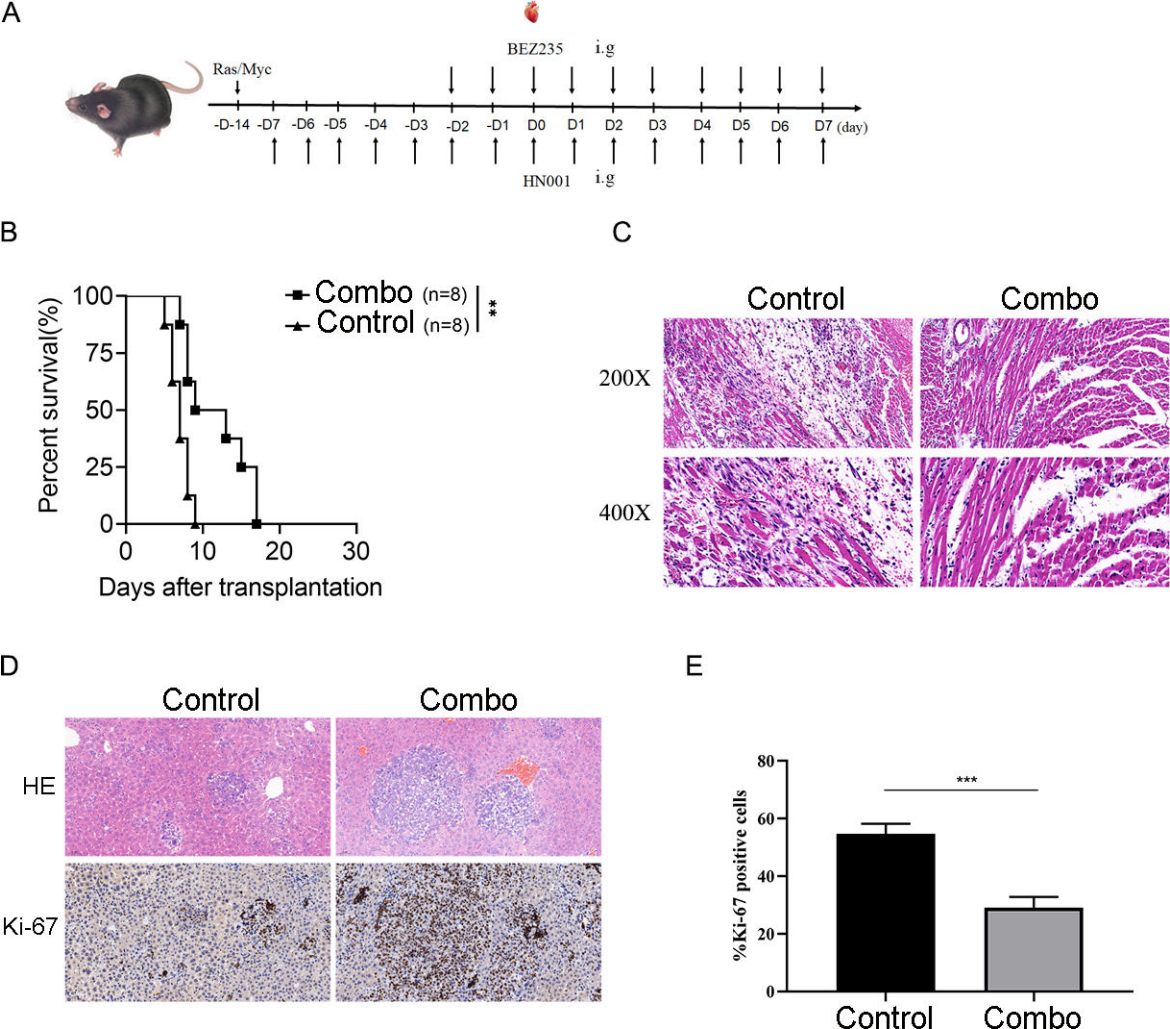
Effect of BEZ235 and *Lactobacillus rhamnosus* HN001 in mice. A schematic diagram is provided to depict the time course of the experiment. (**B**) The survival times of mice that received allografts and underwent combination therapy are compared to those of control mice. (**C**) Histological analysis was conducted to examine the allografts. The heart transplant recipients were sacrificed 7 days after transplantation surgery, and the images were captured at original magnifications of 200× and 400×. (**D**) Histological and immunohistochemical analyses were performed on liver sections with original magnifications of 200× and 400×. H&E staining indicated tumor shrinkage in the combination treatment group, which consisted of BEZ235 and *L. rhamnosus* HN001. The combination treatment also suppressed proliferative activity, as suggested by the reduced proportion of Ki67-positive cells in the tumor areas. (**E**) The percentage of Ki67-positive cells in the tumor areas is presented.

BEZ235, obtained from Selleckchem (Catalog No. S1009), was administered orally via gavage at a dose of 15 mg/kg on the specified dates. *Lactobacillus rhamnosus* HN001 was included as a supplement for mice treated with BEZ235. HN001 was orally administered twice daily via gavage, according to the medication schedule illustrated in Fig. 2A. Our results showed that the combination treatment of BEZ235 and *L. rhamnosus* HN001 significantly prolonged the survival of allografts compared to allograft treatment alone, as shown in Fig. 2B. Histological analysis using H&E staining demonstrated that the allografts from the combination treatment group exhibited infiltration in the myocardium as compared with that in the control group (Fig. 2C). H&E staining showed severe cardiac tissue structural damage and lymphocyte infiltration in the control group. Compared with the control group, the tissue structure of the allogeneic heart transplant in the combination therapy group was relatively intact, and there was also less infiltration of inflammatory cells. Referring to the anticancer efficacy, our therapy showcased a synergistic suppression of primary liver cancer progression in the combination treatment group as opposed to the control group in mice, as depicted in Fig. 2D. Immunohistochemistry evaluation was performed to assess the proliferation of tumor cells in various tissue sections. Representative immunostaining of Ki67 in tumor areas in liver sections to assess the tumour proliferation. Quantification of Ki67+tumor cell numbers per field which represent cells with strong proliferative activity. It was observed that the distribution of Ki67-positive tumor cells was significantly diluted in the combination group compared to other groups. The combinatory treatment comprising of BEZ235 and *L. rhamnosus* HN001 exhibited a remarkable decrease in the percentage of Ki67-positive cells (*P* < 0.001) which indicates suppressed proliferative activity within the tumor area, in comparison to the control as presented in Fig. 2E.

### The combination of BEZ235 and *Lactobacillus rhamnosus* HN001 reduced the production of proinflammatory cytokines

**Fig 3:**
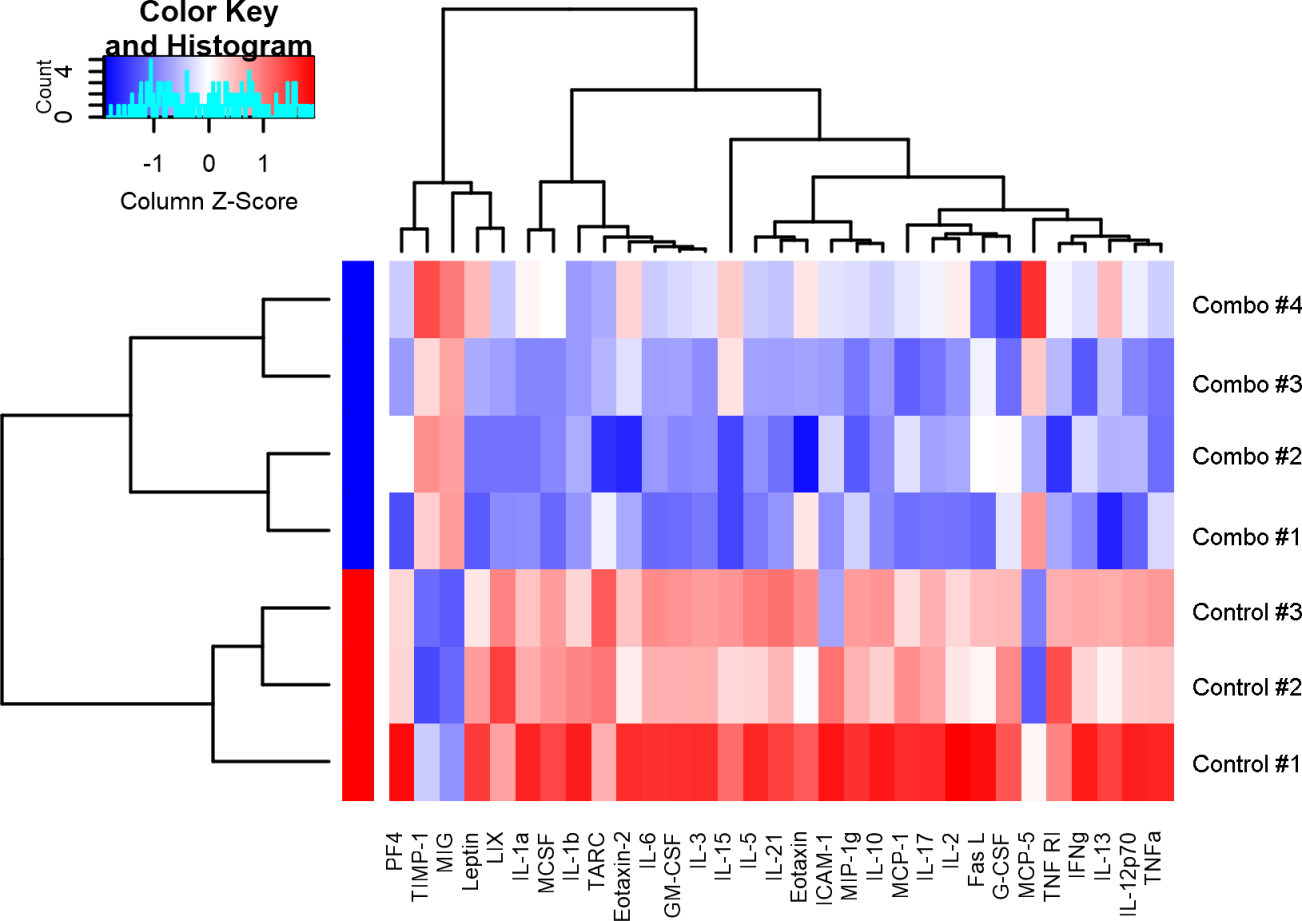
The combination of BEZ235 and *Lactobacillus rhamnosus* HN001 reduced the production of proinflammatory cytokines. The results from the Mouse Inflammation Array Q1 indicated that the cytokine levels in the serum were mainly modified following treatment with BEZ235 and *L. rhamnosus* HN001.

The results of the Mouse Inflammation Array Q1 study revealed the primary modulation of serum cytokine levels upon the administration of BEZ235 and *L. rhamnosus* HN001. Of particular note, the combined treatment resulted in a noteworthy decrease in serum inflammatory cytokine levels in mice with primary liver cancer and as illustrated in Fig. 3.

### The combination treatment regimen is effective by modulating IL-6

**Fig 4:**
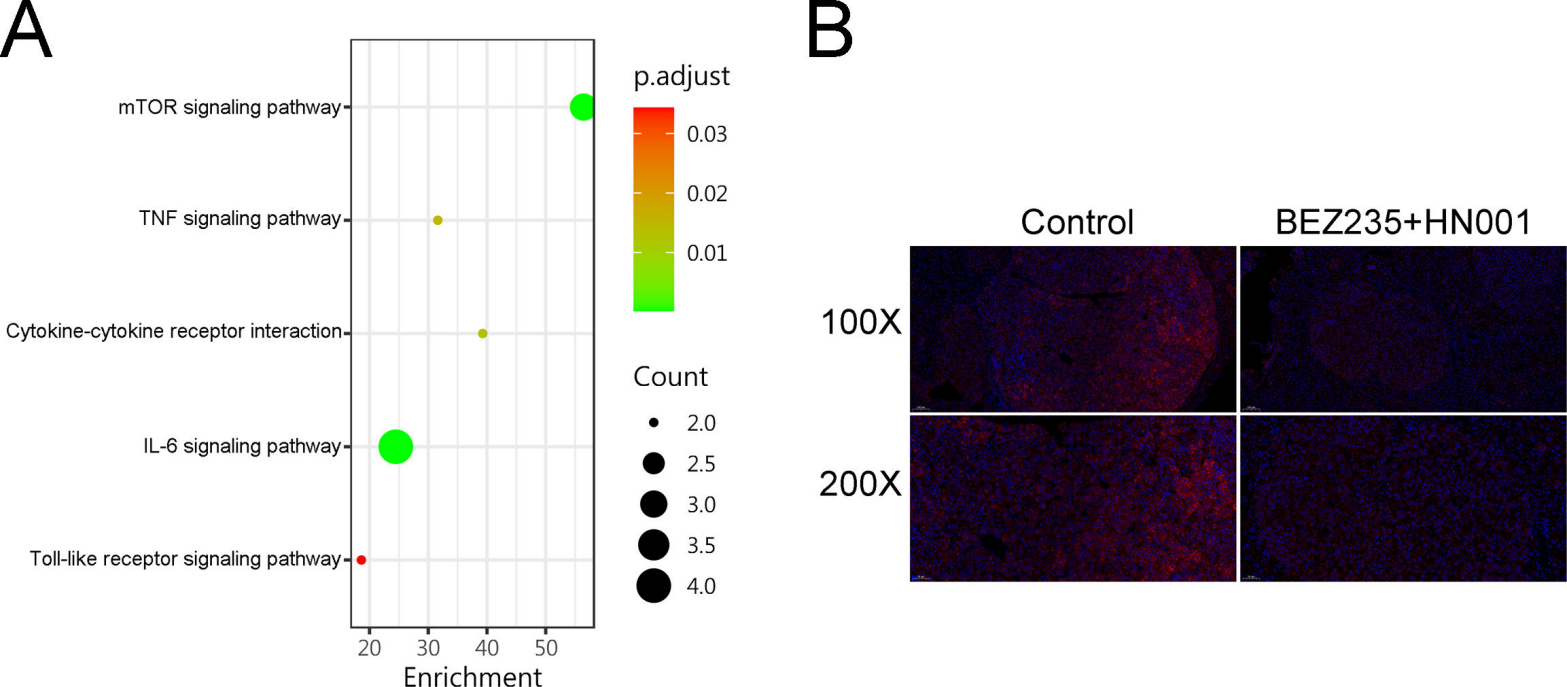
(A) The combination treatment regimen is effective by modulating IL-6. KEGG pathway analysis showing the activated protein in HepG2 cells treated with combination therapy or DMSO for 24 hours. (B) The immunofluorescent staining of liver sections (100× and 200× original magnification) (red: IL-6 immunofluorescence; blue: DAPI).

KEGG pathway analysis revealed a notable impact on the IL-6 signaling pathway, in addition to the mTOR signaling pathway (as demonstrated in Fig. 4A). To further confirm this, protein expression of IL-6 was also analyzed using immunohistochemical staining. As expected, the combined treatment group demonstrated a marked reduction in IL-6 levels compared to the control group, as illustrated in Fig. 4B.

### The combination therapy effectively inhibited the progression of hepatocellular carcinoma after heart transplantation

**Fig 5:**
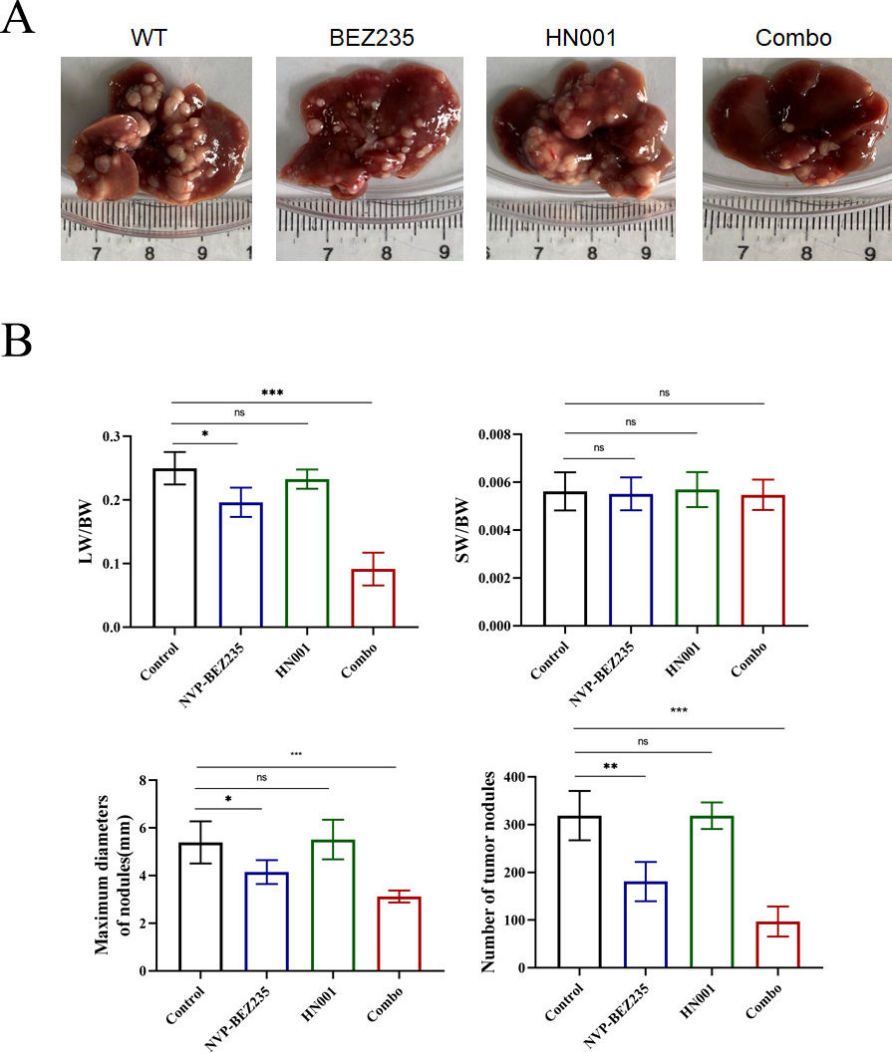
The combination therapy effectively inhibited the progression of hepatocellular carcinoma after heart transplantation. (**A**) Gross images of livers from four mice groups. (**B**) Tumor burdens were calculated by LW/BW ratio, SW/BW ratios, numbers of tumor nodules or maximal diameters. LW/BW, liver weight/body weight; SW/BW, spleen weight/body weight. The data are expressed as the means ± SEM (*n* = 8 per group, NS, *P* ≥ 0.05,*P < 0.05, **P < 0.01, ***P < 0.001) for any other groups versus the control group.

At 4 weeks post-heart transplantation, the mice were sacrificed to undergo phenotypic analysis. Macroscopic evaluation in Fig. 5A demonstrated that the combination therapy of BEZ235 and *L. rhamnosus* HN001 significantly inhibited tumor progression. However, treatment with *L. rhamnosus* HN001 alone failed to improve tumor burdens and exhibited similar liver sizes, maximum tumor diameters, and number of tumor nodules as that of the control group. Although the monotherapy with BEZ235 showed some treatment effectiveness, the combination therapy group exhibited a more pronounced effect. Figure 5B illustrated that the combined treatment significantly ameliorated tumor progression, as measured by LW/BW and SW/BW ratios, maximum tumor diameters, and number of tumor nodules when compared to the control group and either the BEZ235 or *L. rhamnosus* HN001 group. Overall, these data demonstrate the effectiveness of combined treatment with BEZ235 and *L. rhamnosus* HN001 in suppressing hepatocellular carcinoma progression after heart transplantation in mice.

## DISCUSSION

Post-transplant malignancy in heart transplant recipients leads to long-term morbidity and mortality (4, 31–33). Upon initial diagnosis of post-transplant malignancy, 42.3% of cancer cases were found to have extensive or multiple disease status. Despite 88.8% of cases being treated with surgical resection during the initial presentation, almost half (47.3%) experienced progression or recurrence (34). The introduction of advanced immunosuppressive therapy has led to a remarkable improvement in survival rates for individuals who have undergone heart transplantation (35, 36). Nevertheless, an excessive amount of immunosuppression is recognized to heighten the risk of severe infections, renal dysfunction, and cancer development over an extended period (35, 36). Therefore, striking a balance between maintaining adequate immunosuppression to prevent rejection and reducing the likelihood of malignancy formation presents a major challenge in heart transplantation.

mTOR inhibitors have potential advantages in reducing the incidence of post-transplant malignancies and exerting anticancer effects (37). According to reports, mTOR inhibitors have shown clinical benefits in preventing post-transplant malignancies in recipients of cardiac transplants (32). BEZ235, a promising dual inhibitor of PI3K/mTOR, exerts potent antitumor effects by efficiently and selectively blocking the aberrant activation of the PI3K/AKT/mTOR pathway. Through our previous investigation, we have observed that BEZ235 presents a marked advantage over single-target treatments (IC-87114 and rapamycin) in prolonging the survival of transplanted hearts in mice (29). In addition, our subsequent researches have yielded promising results indicating the potential therapeutic effects of BEZ235 on solid tumors (15, 16). However, it has been found that the use of BEZ235 has deleterious effects on the body, particularly in disrupting the intestinal microenvironment. A promising avenue to counteract this may lie in the use of probiotics, as we have observed significant improvements in experimental mouse models that were supplemented with probiotics in conjunction with BEZ235 treatment. In addition to its role in regulating the balance of intestinal flora, probiotics have also been shown to have an immunomodulatory effect that can potentially complement the use of BEZ235 therapy.

As a commercially available probiotic, *L. rhamnosus* HN001 has been found to have a positive effect on the regulation of intestinal flora (28). In order to alleviate the microbial disruptions caused by BEZ235 treatment in mice, we identified *L. rhamnosus* as a potential regulator. This particular strain of probiotic is known for its beneficial effects on gut health (22–24, 27), and the food industry as a therapeutic prob diarrhea (38). Our data demonstrated that supplementation of BEZ235-treated mice with the probiotic *L. rhamnosus* HN001 significantly inhibited the progression of hepatocellular carcinoma after heart transplantation. The results of the study provide evidence that the combination therapy was effective in treating both antitumor immunity and allograft rejection. It was observed that the therapy’s efficacy was closely linked to the regulation of inflammatory IL-6 expression.

IL-6 plays a vital role in both transplantation and tumorigenesis. Increased levels of IL-6 are linked to organ rejection in transplantation (39). Additionally, IL-6 promotes the differentiation of Th17 cells, which may contribute to transplant rejection (40, 41). Hence, effective management of IL-6 levels is critical for successful transplantation. In tumorigenesis, IL-6 promotes tumor growth and metastasis in various cancer types (42–44). It stimulates cancer cell proliferation and survival, as well as angiogenesis (45), and is involved in the development of cancer cachexia, a debilitating wasting syndrome commonly observed in cancer patients (46). Targeting IL-6 shows promise as a therapeutic approach for transplantation and cancer treatment. Blocking IL-6 signaling has been shown to decrease the risk of organ rejection in transplantation (47), while IL-6-targeted therapies are being developed to impede tumor growth and enhance patient outcomes in cancer treatment (48). Further research into the role of IL-6 in transplantation and tumorigenesis may lead to novel approaches for managing these ailments.

The combination of BEZ235 with other chemotherapeutic agents significantly enhanced the efficacy of drug therapy or alleviated side effects, thereby overcoming drug resistance (49, 50). A notable increase in the expression of IL-6 in Hepatocellular carcinoma (HCC) cells was observed in our previous research, indicating that the administration of BEZ235 could potentially induce an inflammatory response within the body (15). The development of tumors is closely associated with the occurrence of inflammatory reactions. Cytokines are regarded as the crucial mediators that link inflammation and cancer (51). Playing a vital role as the central cytokine within the body, IL-6 also participates in regulating the immune response within the tumor microenvironment and promoting tumor proliferation (52). A considerable quantity of clinical samples has demonstrated that patients with HCC have significantly elevated serum IL-6 levels compared to healthy individuals, and these high levels of serum IL-6 are associated with a unfavorable prognosis (53). According to our data, the addition of *L. rhamnosus* HN001, a probiotic, effectively suppressed the expression of IL-6 in BEZ235-treated mice. This effectively mitigates the unfavorable effects of BEZ235, thereby leading to enhanced treatment efficacy. Consequently, the co-administration of *L. rhamnosus* HN001 and BEZ235 could potentially amplify the inhibitory efficacy of BEZ235 in impeding the progression of HCC. This could potentially be the mechanism behind the synergistic effect.

In summary, the present study provides a novel and efficient therapeutic immunotherapy for solid organ transplant recipients. We demonstrate that this novel immunotherapy is potent and safe in the treatment of transplanted animals with established primary liver cancer and prolonged the survival of allograft. These findings have important clinical implications for understanding the balance between antitumor immunity and allograft rejection.

## MATERIALS AND METHODS

### Animals

Male C57BL/6 (B6; H-2b) and BALB/c (B/c; H-2d) mice (8 weeks of age) were procured from Beijing Vital River Laboratory Animal Technology Co., Ltd. (Beijing, China). The animal study was authorized by the Institutional Animal Care and Use Committee (IACUC) of Zhejiang University, and to ensure consistent environmental conditions, all mice were reared in a specific-pathogen-free environment with free access to food and water, and subjected to a regular 12 hour light/dark cycle. The research protocols were sanctioned by the IACUC at Zhejiang University (Zhejiang, China).

For the probiotics experiment, the mice were randomly assigned to four groups: the control group (*n* = 8), the BEZ235 group (*n* = 8), the probiotics group (*n* = 8), and the combined group (BEZ235 with 2 × 10^8^ colony-forming units of *L. rhamnosus* HN001, *n* = 8). The *L. rhamnosus* HN001, provided by Nutrition & Biosciences, DuPont, was suspended in 200 µL of phosphate buffer saline and administered orally to the BEZ235-treated mice via gavage twice daily for 2 weeks.

### Vascularized heterotopic cardiac transplantation

Vascularized heterotopic models of heart transplantation were constructed following established procedures (54). In brief, the hearts from male C57BL/6 donors (B6; H-2b) were transplanted into the subcutaneous region of the right neck of male BALB/c recipient mice (B/c; H-2d). The cardiac grafts and peripheral blood were subsequently harvested.

### Inflammatory array

The concentrations of inflammatory mediators were assessed using a Mouse Cytokine Array QAM-INF-1–2 (RayBiotech) coated with 40 distinct cytokines as per the protocol provided by the manufacturer. In summary, the arrays were initially blocked and then incubated overnight with 100 mL of conditioned medium. This was followed by treatment with a biotin-conjugated antibody (1/250) for 2 hours. Subsequently, the membranes were treated with a peroxidase-based substrate, and the results were recorded utilizing XAR films. Quantitative analysis of the data was then performed with the aid of Array Vision Evaluation 8.0 (GE Healthcare Life Science).

### Hematoxylin and eosin staining (H&E)

After 7 days of transplantation, cardiac grafts and liver samples were collected. The collected samples were cross-sectioned and subjected to fixation in 10% formalin (SF98-4; Fisher) at 4°C until further use. Next, the fixed tissues were dehydrated, embedded in paraffin, and sliced into 5-µm sections. Finally, H&E staining was carried out.

### Immunohistochemical staining (IHC) and immunofluorescence

In brief, following deparaffinization and rehydration, sections were subjected to heat in citrate buffer at 121°C for 30 minutes. Then, they were treated with 0.3% hydrogen peroxide in methanol for 20 minutes, blocked with 10% normal bovine serum, and incubated overnight with rabbit polyclonal antibodies at 4°C. After the primary antibody incubation, slides were treated with Alexa Fluor-conjugated secondary antibody (Life Technologies) diluted in block buffer for 1 hour at room temperature. Finally, slides were examined using a laser scanning confocal microscope (Zeiss LSM 800).

### Statistical analysis

The experimental data were analyzed using SPSS v23 (SPSS Inc., Chicago, IL). The normality and equal variance tests were performed on the data, and at least three independent experiments were conducted. The sample size was calculated using PASS 11 (NCSS Inc.). For statistical analyses, Student’s *t*-test was used to compare between two groups, while one-way analysis of variance (ANOVA) followed by Bonferroni’s post-hoc test was employed

for other comparisons. All results were expressed as mean ± standard error of the mean (SEM). All statistical tests were considered two-tailed, and a *P*-value of less than 0.05 was considered statistically significant.

## Data Availability

All data generated in the study are included in this article. Raw sequence data are accessible on the NCBI platform under accession no.PRJNA836559.

## AFTER EPUB

*[This article was published on 2 April 2024 with an error in the author affiliations. The author affiliations were corrected in the current version, posted on 15 April 2024.]*
